# Forensic Utility of Vitreous Potassium Concentration in Estimating Postmortem Interval: A Systematic Review

**DOI:** 10.7759/cureus.108128

**Published:** 2026-05-02

**Authors:** Remya Sadananda Shenoi, Anu Sasidharan, Ajay Balachandran

**Affiliations:** 1 Forensic Medicine and Toxicology, Amrita School of Medicine, Amrita Institute of Medical Sciences and Research Centre, Amrita Vishwa Vidyapeetham, Kochi, Ernakulam, IND

**Keywords:** pmi, postmortem interval, thanatochemistry, vitreous humor, vitreous potassium

## Abstract

Estimation of postmortem interval (PMI) is a fundamental component of forensic pathology. One of the reliable methods is assessment using biochemical markers such as sodium, potassium, chloride, bicarbonate, and hypoxanthine. Among biochemical markers, vitreous potassium (K⁺) has been extensively studied due to its predictable postmortem changes and relative stability. In comparison to other body fluids, vitreous humor is resistant to early putrefaction changes and is least affected by external factors. A systematic search of PubMed and Scopus databases was conducted using predefined search strategies. Studies involving human subjects, measurement of vitreous potassium, and evaluation of its relationship with PMI were included. A total of 123 studies were identified from PubMed and Scopus; 24 studies met the inclusion criteria and were included. Vitreous potassium demonstrated a strong positive correlation with PMI (r = 0.84-0.99), with most studies supporting a linear relationship within the first 72 hours, although some reported a biphasic pattern. Significant variability in regression models was observed due to differences in environmental conditions, analytical methods, and study populations. Temperature showed inconsistent but notable effects, particularly at extended PMIs, where deviations from linearity reduced reliability. Methodological heterogeneity further influenced results, while recent evidence suggests improved accuracy when potassium is combined with additional biochemical markers such as hypoxanthine and albumin. Vitreous potassium is a reliable marker for estimating PMI, especially in early stages. However, variability limits its standalone accuracy, and integration with multiparameter models is recommended.

## Introduction and background

Estimation of the postmortem interval (PMI) remains one of the most challenging aspects of forensic practice. After death, bodies undergo various decomposition changes, both physical and chemical. For greater precision in estimating time of death and for scientific understanding, the electrolyte composition of biological fluids has been the main focus of research in forensic medicine. Traditional methods of calculating time since death by assessing rigor mortis, livor mortis, and algor mortis are inherently subjective and are influenced by environmental and individual factors [1-3]. Consequently, biochemical approaches have gained increasing importance in forensic thanatology [4]. Vitreous humor is considered an ideal medium for postmortem biochemical analysis due to its anatomical isolation, resistance to putrefaction, and relatively stable composition [5,6]. Among various biochemical markers, potassium has emerged as the most extensively studied parameter and is widely regarded as the most reliable single biochemical indicator for PMI estimation [7,8].

Correlation between potassium levels and PMI was initially reported by Sturner and Gantner [9] and Adelson et al. [10]. Following death, loss of cellular membrane integrity results in passive diffusion of intracellular potassium into the extracellular vitreous humor, leading to a progressive increase in concentration over time [9,11]. This phenomenon has been consistently demonstrated across multiple studies, forming the basis for regression models correlating vitreous potassium levels with PMI [12-14]. Despite its widespread application, variability in regression equations and influencing factors such as temperature, cause of death, and analytical methods have raised concerns regarding its universal applicability [15-17]. Recent advances have focused on improving accuracy through multiparameter approaches and integration with modern analytical techniques [18-20].

## Review

Materials and methods

A systematic search was conducted in accordance with the PRISMA 2020 guidelines [21]. A literature search was conducted in the PubMed and Scopus databases on February 26, 2026. The search strings used for the PubMed and Scopus databases are as follows: PubMed: ("Vitreous Humor"[MeSH Terms] OR vitreous) AND potassium AND ("postmortem interval" OR "time since death") and Scopus: ("vitreous potassium" OR "vitreous humour potassium" OR "vitreous humor potassium") AND ("postmortem interval" OR "time since death" OR PMI).

A total of 123 articles were identified; 56 from Scopus and 67 from the PubMed database. After removal of duplicates and screening for inclusion and exclusion criteria, 24 studies were included in the current systematic review. Only human studies published in English were included in this review. Original research studies in which vitreous potassium concentrations were measured and the relationship between potassium and PMI was evaluated were included in this analysis. Original studies on animals, case reports, and studies without sufficient correlation or regression data were excluded.

Data Extraction

Data extracted included (1) author(s); (2) year of publication; (3) country/countries where the study was conducted; (4) study design; (5) sample size; (6) PMI range; (7) temperature considerations; (8) analytical methods; (9) regression equations/correlation coefficients; and (10) key findings. Data extraction was performed by a single reviewer using the data extraction table with the details mentioned above over a period of 15 days. The extracted data were cross-checked for accuracy by a separate reviewer.

Due to heterogeneity in study designs, analytical techniques, and environmental conditions, a quantitative meta-analysis was not conducted. The findings were therefore synthesized qualitatively. The methodological quality of the included studies was assessed using the Joanna Briggs Institute critical appraisal checklists [22], appropriate to the study designs, and is presented in the Risk of bias assessment section. The checklist items were grouped into domains of selection bias, measurement bias, and confounding for simplified interpretation, and an overall risk of bias was assigned for each study.

**Figure 1:**
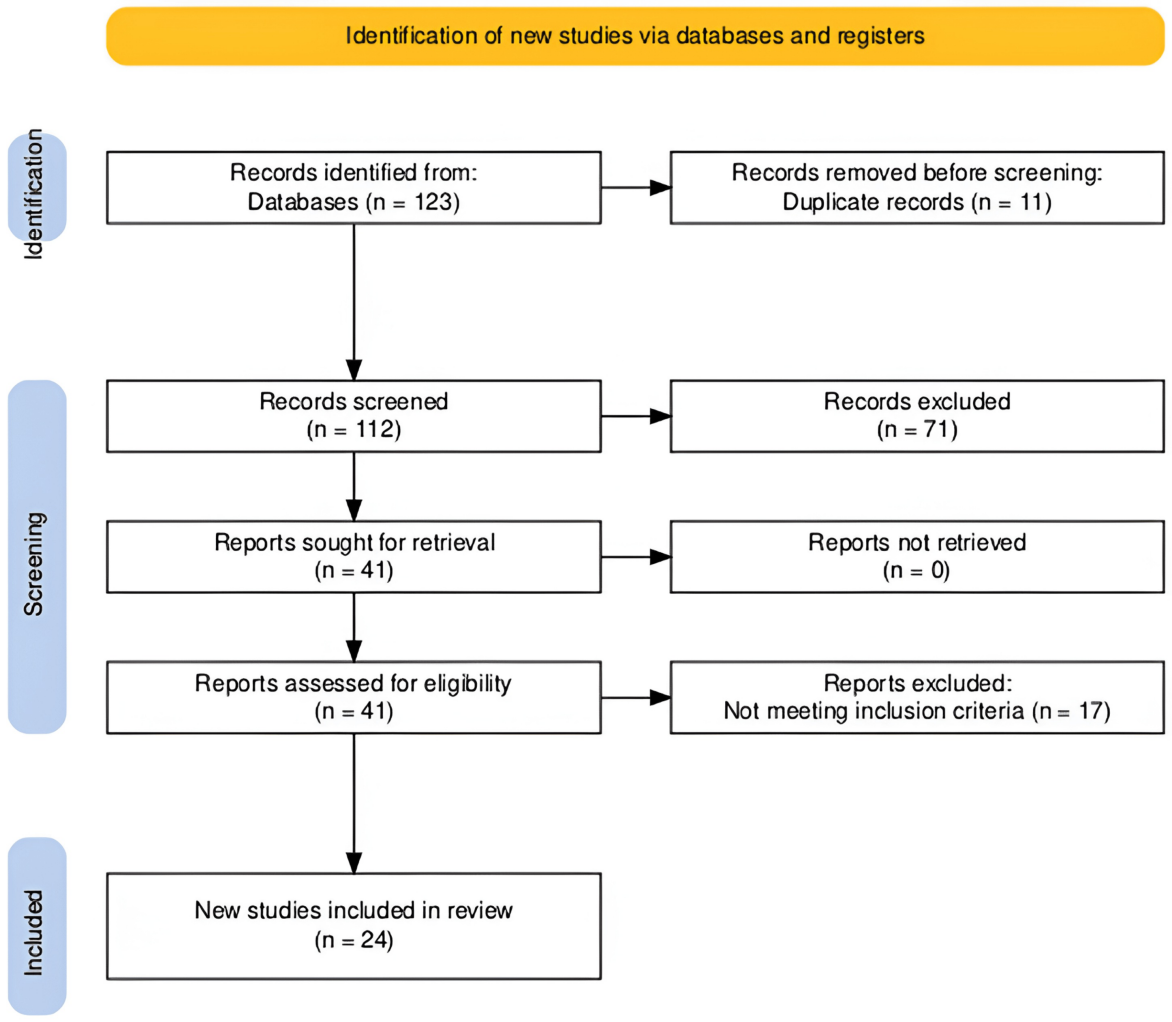
PRISMA flow diagram of study selection

The study selection process is illustrated in Figure 1.

Relationship between vitreous potassium and PMI

All included studies demonstrated a progressive increase in vitreous potassium concentration with increasing PMI. The majority reported a linear relationship, particularly within the early postmortem period (<72 hours) [9,11]. While most studies demonstrate a linear increase in vitreous potassium with increasing PMI, some suggest a biphasic pattern, with a steeper rise in the early postmortem period followed by a slower rate of increase at later intervals [4,11,23,24]. The exact nature of this relationship remains unsettled. Reported correlation coefficients ranged from r = 0.84 to 0.99, indicating a strong association across studies. However, regression equations varied widely, with slopes ranging approximately from 1.0 to 7.1 hours per mmol/L. This wide variation reflects differences in environmental conditions, analytical techniques, and study populations. Several studies reported deviation from linearity at prolonged PMIs (>100 hours), suggesting reduced reliability of potassium-based models in late postmortem periods [17].

Effect of temperature

Temperature was identified as a major influencing factor. Zilg et al. [11] evaluated late PMIs (up to 17 days) in bodies stored in mortuary cold rooms and observed a nonlinear rise in vitreous potassium. Decedent age and ambient temperature contributed to variability by 16% and 5%, respectively. In contrast, Foster et al. [25] did not account for environmental temperature. Earlier, Sturner and Gantner [9] suggested that temperature does not significantly influence vitreous potassium levels. However, Komura and Oshiro [12] reported a decline in potassium concentration in human and rabbit eyes at low temperatures. Similarly, Rognum et al. [7] demonstrated variation in potassium elevation across four distinct environmental temperatures. These findings highlight the inconsistent influence of temperature on vitreous potassium, underscoring its role as a potential confounding factor in PMI estimation.

Methodological variability

Different analytical techniques were used across studies to measure vitreous potassium ion concentration, including flame photometry, ion-selective electrodes, and advanced biosensor methods. These variations contributed significantly to differences in reported regression equations [16]. Bortolotti et al. [26] utilized capillary ion analysis with UV detection; however, this method demonstrated limited sensitivity and required complex instrumentation. Zhou et al. [20] measured vitreous potassium using low-pressure ion chromatography but were unable to adequately account for qualitative interference from other components. Passos et al. [6] described a sequential injection system for automated detection of potassium and hypoxanthine; however, the method involved complex pretreatment steps and prolonged operational time. These methodological variations may contribute to inter-study differences and represent a potential source of measurement bias. Recent studies demonstrated improved accuracy using combined models incorporating potassium along with other biochemical markers such as hypoxanthine and albumin [18,19].

The characteristics of included studies are summarized in Table 1.

**Table 1:** Summary of data extracted from the studies included in the systematic review PMI, postmortem interval

S. no.	Author	Year	Country	Study design	Sample size	PMI range	Temperature considered	Method	Regression equation/correlation	Main findings
1	Sturner and Gantner [9]	1964	USA/UK	Observational	~91	Up to 100 hours	Not standardized; environmental variation	Flame photometry	PMI = 7.14 × K - 39.1 (r = 0.987)	Strong linear correlation
2	Komura and Oshiro [12]	1977	Japan	Experimental	90	Not specified	Environmental temperature studied	Flame photometry	Not specified	Temperature influences K⁺
3	Balasooriya et al. [13]	1984	UK	Observational	59	Not specified	Temperature-dependent changes	Autoanalyzer	Linear relationship	Inter-eye variability noted
4	Madea et al. [14]	1990	Germany	Observational	100	Not specified	Ambient temperature affects slope	Regression comparison	Multiple regression formulas; PMI = 5.26 × K - 30.9; slope 0.17-0.19 mmol/L/hour	Formula variability; temperature affects slope
5	Gamero Lucas et al. [15]	1992	Spain	Comparative	Not specified	Not specified	Temperature, age, terminal phase duration	Equation comparison	Regression demonstrated	High inter-study variability
6	Lange et al. [16]	1994	USA	Pooled analysis	790	Variable	Environmental + biological factors	Statistical modeling	Nonlinear relationship	Not strictly linear across datasets
7	Tagliaro et al. [17]	1999	Italy	Method + observational	20	5-96 hours	Influencing factor	Capillary electrophoresis	K = 0.215 PMI + 4.755 (r = 0.904)	Reliable analytical method
8	Myo-Thaik-Oo et al. [18]	2002	Japan/Myanmar	Observational	82	1-60 hours	Temperature considered	Blood gas analyzer	PMI = 5.26 × K - 6.01; PMI = 5.32 × K - 4.93 (r = 0.93)	Strong correlation
9	Muñoz Barús et al. [19]	2002	Spain	Observational	206	Not specified	Not specified	Regression analysis	PMI = 3.967 × K - 19.186	Cause of death improves accuracy
10	Zhou et al. [20]	2007	China	Observational	62	1-27 hours	Environmental influence	Ion chromatography	K = 0.1702 PMI + 5.5678 (r = 0.8692)	Linear correlation
11	Swain et al. [23]	2015	India	Observational	100	<96 hours	Stored at 4-8°C	Autoanalyzer	PMI = -11.86 + 2.88 × K	Vitreous humor better than CSF
12	Foster et al. [25]	2016	UK	Observational	78	6-62 hours	Temperature considered	Ion-selective electrode	PMI = 6.42 × K - 40.94	Moderate correlation
13	Bortolotti et al. [26]	2011	Italy/USA	Observational	164	2-110 hours	Not specified	Capillary ion analysis	K = 0.1733 PMI + 2.3008 (r = 0.962)	Strong correlation
14	Jashnani et al. [27]	2010	India	Prospective	120	Not specified	No significant effect	Flame photometry	PMI = 1.076 × K - 2.815	Differs from Western data; K⁺ rises
15	Mihailovic et al. [28]	2012	Serbia	Experimental	320 samples (32 cases)	≤30 hours	Controlled 20°C	Repeated sampling	PMI = 2.749 × K - 11.978	Controlled conditions improve accuracy
16	Deokar and Shendarkar [29]	2013	India	Observational	152	1.6-39.2 hours	Not specified	Biochemical analysis	Linear relationship	Strong correlation
17	Siddamsetty et al. [30]	2014	India	Cross-sectional	210	>72 hours	Climate considered	Biochemical analysis	PMI = 4.701 × K - 29.603 (r = 0.841)	Strong correlation
18	Bohra et al. [31]	2014	India	Observational	200	0-72 hours	No significant effect	Ion-selective electrode	PMI = -16.22 + 3.75 × K	No effect of age/sex/temperature
19	Rathinam et al. [32]	2015	India	Cross-sectional	55	0 to >24 hours	No significant effect	Ion-selective electrode	1 mEq/L increase = 1.99 h PMI	Valid in trauma cases
20	Dhanak and Johry [33]	2016	India	Prospective	200	Early PMI	Not specified	Ion-selective autoanalyzer	1 mEq/L increase = 2.99 h PMI	Strong early PMI relation
21	Ortmann et al. [34]	2016	Germany	Observational	600	0-125 hours	Ambient + biological factors	Five-equation comparison	Slope 0.17-0.19 mmol/L/hour	High variability
22	Ding et al. [35]	2017	China	Experimental	63	≤36 hours	Not specified	Fluorescence aptasensor	PMI = -0.55 + 1.66 × K (r = 0.791)	High sensitivity, not clinically validated
23	Cordeiro et al. [36]	2019	Portugal	Observational	331	Not specified	Ambient + rectal temp + weight	Indirect potentiometry	Multivariable additive models	Combined variables improve accuracy
24	Paul et al. [37]	2021	India	Prospective	75	4 hours and 50 minutes to 36 hours and 40 minutes	Not specified	Ion-selective automated analyzer	PMI = 1.075 × K - 2.53 (r = 0.997)	Very strong correlation

Risk of bias assessment

The risk of bias assessment demonstrated that the majority of included studies had a moderate overall risk of bias. Selection bias was generally low to moderate across studies, while measurement bias was predominantly low due to the use of standardized biochemical techniques. However, confounding factors, particularly environmental and storage temperature, were inadequately addressed in several studies, contributing to a higher risk of bias in this domain. Only a few studies employed controlled conditions or multivariable models to minimize confounding effects. Overall, five studies were categorized as low risk, while the remaining studies were predominantly of moderate risk, with no studies demonstrating a high overall risk of bias. Confounding due to temperature variation was the most frequently identified source of bias across studies. The observed heterogeneity in results, particularly related to temperature and methodological variability, is consistent with the identified confounding and measurement biases across studies.

Risk of bias assessment is summarized in Table 2.

**Table 2:** JBI risk of bias assessment summary JBI, Joanna Briggs Institute

S. no.	Author (year)	Selection bias	Measurement bias	Confounding (temperature, methodology, etc.)	Overall risk
1	Sturner and Gantner (1964) [9]	Moderate	Moderate	High	Moderate
2	Komura and Oshiro (1977) [12]	Low	Moderate	Low	Low
3	Balasooriya et al. (1984) [13]	Moderate	Moderate	Moderate	Moderate
4	Madea et al. (1990) [14]	Low	Low	Moderate	Low
5	Gamero Lucas et al. (1992) [15]	Moderate	Moderate	Moderate	Moderate
6	Lange et al. (1994) [16]	Low	Moderate	Moderate	Moderate
7	Tagliaro et al. (1999) [17]	Moderate	Low	Moderate	Moderate
8	Myo-Thaik-Oo et al. (2002) [18]	Low	Low	Low	Low
9	Muñoz Barús et al. (2002) [19]	Low	Moderate	High	Moderate
10	Zhou et al. (2007) [20]	Moderate	Low	Moderate	Moderate
11	Swain et al. (2015) [23]	Moderate	Moderate	Low (controlled storage conditions)	Moderate
12	Foster et al. (2016) [25]	Moderate	Low	Low	Moderate
13	Bortolotti et al. (2011) [26]	Low	Low	High	Moderate
14	Jashnani et al. (2007) [27]	Low	Moderate	Moderate	Moderate
15	Mihailovic et al. (2012) [28]	Low	Low	Low	Low
16	Deokar and Shendarkar (2013) [29]	Moderate	Moderate	High	Moderate
17	Siddamsetty et al. (2014) [30]	Low	Moderate	Low	Moderate
18	Bohra et al. (2014) [31]	Moderate	Moderate	High	Moderate
19	Rathinam et al. (2015) [32]	Moderate	Moderate	High	Moderate
20	Dhanak and Johry (2016) [33]	Low	Moderate	High	Moderate
21	Ortmann et al. (2016) [34]	Low	Moderate	Moderate	Moderate
22	Ding et al. (2017) [35]	Low	Low	High	Moderate
23	Cordeiro et al. (2019) [36]	Low	Low	Low	Low
24	Paul et al. (2021) [37]	Low	Low	High	Moderate

Discussion

The present systematic review confirms a consistent increase in potassium concentration with increasing PMI, particularly within early PMIs [9,12-14]. However, there is no consensus regarding the mathematical equation that best explains this increase. Existing models generally assume a linear increase in vitreous potassium with PMI; however, variations in slope and baseline values have been reported [11]. Thus, this limits the universal applicability of potassium as a standalone marker. Harper [38] noted that vitreous humor samples rarely contain significant numbers of bacteria or fungi, rendering them less susceptible to postmortem solute changes compared to other body fluids.

A major limitation is the significant variability in regression equations, which has been highlighted in multiple comparative and analytical studies [15-17,26-28]. Differences in study populations, environmental conditions, and analytical techniques contribute to inconsistencies in slope and intercept values, limiting the generalizability of any single predictive model.

Temperature is an important influencing factor, although its effect has been inconsistently reported across studies. Both experimental and observational studies have demonstrated that ambient and storage temperatures significantly affect the rate of potassium increase [29-31]. Cooling delays biochemical changes, whereas higher temperatures accelerate them, emphasizing the need for temperature-adjusted models. Sturner and Gantner [9] reported that temperature does not significantly influence vitreous potassium concentrations. However, this finding contrasts with the longstanding view that potassium levels are affected by ambient temperature, consistent with the general principle that biological processes are slowed at lower temperatures.

Methodological variability also plays a substantial role. Differences in analytical techniques such as flame photometry, ion-selective electrodes, and capillary electrophoresis contribute to inter-study variation [23,25,32]. Even minor variations in sample handling and calibration can influence measured potassium levels.

Another important observation is the nonlinear behavior of potassium at extended PMIs. Several studies have reported plateauing or a reduced rate of increase beyond 100 hours, limiting their reliability in late PMIs [33-35]. Ortmann et al. [34] used five different equations to assess the time since death in 600 cases where the exact time since death was known. They observed that the standard deviations between real and extrapolated time since death increase with increasing PMI or potassium concentration. Improved precision may be achieved by multiple linear regression analysis, incorporating not only the potassium concentration but also other vitreous humor electrolytes.

Biological factors such as cause of death and metabolic state may also influence potassium levels. Some studies demonstrated improved predictive accuracy when such variables were incorporated into regression models [36]. In a study by Zilg et al. [11], it was observed that the age of the deceased significantly influenced the rise in potassium concentrations, with younger individuals demonstrating a more rapid increase. The differences were observed across every age group. Most of the studies did not consider age as a factor that can cause significant changes in vitreous potassium concentration. Recent research highlights a shift toward multiparameter models, combining potassium with other biochemical markers such as hypoxanthine and albumin [3,24,37]. These approaches have shown improved accuracy and represent the future direction of PMI estimation.

Despite these limitations, vitreous potassium remains a simple, cost-effective, and widely applicable tool in forensic investigations. However, it should always be interpreted in conjunction with other findings for optimal accuracy. Limitations of the study include heterogeneity in study design and methodology, variability in temperature conditions, limited data for late PMIs, lack of standardized regression models, and potential publication bias. Language restriction (English-only studies) may have influenced the findings.

## Conclusions

Vitreous potassium is a valuable biochemical marker for estimating PMI, particularly in early postmortem periods. However, variability in influencing factors limits its standalone accuracy. Future research should focus on standardization and integration of multiparameter models. The wide variation in regression slopes (approximately 1.0-7.1) across studies likely reflects differences in environmental conditions, study populations, and analytical methodologies, thereby limiting the universal applicability of vitreous potassium-based PMI estimation.
